# Chicago Society of Physicians and Surgeons

**Published:** 1875-10

**Authors:** E. Warren Sawyer


### CHICAGO SOCIETY OF PHYSICIANS AND SURGEONS

*Regular Meeting, Aug.* 23, 1875.

(Reported by E. WARREN SAWYER, M.D.)

President, Dr. Bevan, in the chair.

The Society listened with great interest to an exhaustive paper on the etiology, pathology and treatment of the intestinal affections of children, by Prof. N. S. Davis.

In the discussion of Dr. Davis's paper, Dr. Paoli remarked that he used vegetable astringents, and especially quinine, with great circumspection in children, because of the danger of inducing cerebral hyperæmia. He extolled, with Dr. Davis, the use of the mineral acids, and spoke of the efficacy of port wine.

Dr. Bartlett remarked, that in relying upon the temperature record, given by the Signal Service Bureau, it is important to remember that this record is taken at an altitude of from fifty to seventy feet, and that the temperature at that height is, at least, ten degrees lower than that upon the earth; he gave several examples of disparity between the temperature low down and that reported by the signal service.

Dr. Hamill remarked that there must be some other exciting cause of the bowel troubles of children beside heat; if not, why are these diseases confined almost exclusively to populous districts?

Dr. Hyde asked to respond to Dr. Hamill. There is another cause than heat, else the children in the South, and in Africa, would be especially prone to bowel troubles which, by no means, is the case. Heat probably arouses other causes. In this connection it is interesting to refer to a series of tables made in this city not long since, showing that in each ward of the city the infant mortality from bowel affections was in direct ratio to the extent of sewerage possessed by that ward; the ward most imperfectly drained, had the greatest mortality. It is important, too, to examine into the supposed miasmatic origin of these bowel affections; a medical gentleman, of extended observation, had told him that in San Francisco he regarded the bowel troubles of children as wholly of miasmatic origin, and gave quinine freely in these cases. Practitioners on the continent are less apprehensive concerning the bowel affections of children than we are, and they exhibit almost a fear in the administration of quinine.

Dr. Davis remarked that we may yet learn that, besides the continuous heat, there are other important atmospheric conditions that bear directly upon the causation of the bowel troubles of children; thus, still air, little winds, or the degree of moisture. He did not regard these affections as of malarial origin; if this were true, then there would be a relation between the bowel affections and the presence of malaria; the bowel troubles would be most common when malarial affections are most common, which is not the case. He thought the statement, that cholera infantum was a disease of populous districts, would not hold good; it is found everywhere.

Dr. Simon confirmed the statement of Dr. Hyde, respecting the use of quinine in Paris, and said that it was almost never given there to children. The successful results in infant bowel troubles, in Paris, could be attributed, first, to the free use of alcholic stimulants; second, to the administration of raw meat, given in pills at short intervals.

Dr. Fisher read to the Society the report of a case of dislocation of the hip, which was reduced by manipulation sixteen days afterward. The patient was a little girl who lived in Iowa. She jumped from the height of three feet, and fell; she was picked up vomiting, and could not stand. Medical attendance was summoned, and the lesion was treated for rheumatism. Another attendant prescribed a liniment for a supposed hip disease. Dr. Fisher asked to have the girl brought to this city, and he saw her sixteen days after the injury. It had the typical characteristics of a dislocation of the head of the femur upon the dorsum ilii; shortening of the extremity; great toe of the injured side crossing the opposite instep, and finally the head of the femur was felt upon the dorsum of the ilium. Dr. Hyde saw the patient, and at once confirmed the diagnosis. The patient was etherized by Dr. Hyde, when Dr. Fisher, by Reid's method of manipulation, returned the dislocated head of the femur to its socket so easily and noiselessly, that he hardly knew when it was reduced.

Dr. Fisher remarked that this was the third instance in which he had reduced a dislocated hip by Reid's method, and always with the utmost facility.

